# Factors that affect the growth and photosynthesis of the filamentous green algae, *Chaetomorpha valida*, in static sea cucumber aquaculture ponds with high salinity and high pH

**DOI:** 10.7717/peerj.6468

**Published:** 2019-02-14

**Authors:** Ronglian Xing, Weiwei Ma, Yiwen Shao, Xuebin Cao, Lihong Chen, Aili Jiang

**Affiliations:** 1 College of Life Sciences, Yantai University, Yantai, China; 2 National Algae and Sea Cucumber Project Technology Research Centre, Shandong Oriental Ocean Sci-Tech Co., LTD, Yantai, China

**Keywords:** Filamentous green algae, *Chaetomorpha valida*, Growth, Photosynthesis, Salinity, pH, Sea cucumber pond

## Abstract

*Chaetomorpha valida*, dominant filamentous green algae, can be harmful to sea cucumber growth in aquaculture ponds of China. In order to understand the environmental factors affecting the growth of *C. valida* in sea cucumber aquaculture ecosystems, a combination of field investigations and laboratory experiments were conducted. Field surveys over one year revealed that *C. valida* survived in sea cucumber aquaculture ponds in salinities ranging from 24.3 ± 0.01‰ to 32.0 ± 0.02‰ and a pH range of 7.5 ± 0.02–8.6 ± 0.04. The high salinity and pH during the period of low *C. valida* biomass from January to May lay the foundation for its rapid growth in the following months of June to October. Many factors interact in the field environment, thus, laboratory experiments were conducted to determine the isolated effects of pH and salinity on *C. valida* growth. In laboratory experiments, samples were incubated under different salinity and pH conditions at 25 °C, with a light intensity of 108 μmol photon·m^−2^·s^−1^, and a photoperiod of 12 L:12 D. Results showed that salinity and pH significantly affect the growth and *F*_v_/*F*_m_ (quantum yield of photosynthesis) of *C. valida* (*p* < 0.01). *C. valida* grew the longest at a salinity of 34‰ and a pH of 8.0. At 34‰ salinity, *C. valida* grew to 26.44 ± 5.89 cm in 16 days. At a pH of 8.0, *C. valida* grew to 67.96 ± 4.45 cm in 32 days. *F*_v_/*F*_m_ was 0.635 ± 0.002 at a salinity of 32‰, and 0.550 ± 0.006 to 0.660± 0.001 at pH 7.0 to 8.5. Based on these results, we conclude that *C. valida* can bloom in sea cucumber ponds due to the high salinity and pH of coastal sea waters, which promote growth and maintain the photosynthetic activity of *C. valida*.

## Introduction

The Green tide is one of the most important issues affecting shallow coastal areas around the world (Edlund et al., 2009; Bianchi et al., 2010; Harmon et al., 2014; Fleming-Lehtinen et al., 2015). Outbreaks of thalloid green macroalgae (e.g., *Ulva armoricana* and *Ulva rotundata*) occur near the Yellow sea coast of China. However, outbreaks of filamentous green macroalgae (e.g., *Chaetomorpha* spp. and *Cladophora* spp.) are found in ponds and lakes (Le Luherne et al., 2016). The dominant species of filamentous green macroalgae are in the genera *Ulva*, *Chaetomorpha*, *Cladophora*, *Enteromorpha*, and *Codium*, which are characterized by rapid absorption of nutrients and high growth rates (Valiela et al., 1997; Anderson, Glibert & Burkholde, 2002; Zhang & Shao, 2013). In recent years, an invasive filamentous green algae, *Chaetomorpha valida*, has been found in sea cucumber aquaculture ponds (Chi et al., 2009; Deng et al., 2012).

*Chaetomorpha* is a common and widespread green algae genus in the order Cladophorales which is characterized by unbranched filaments. *C. valida* was originally described from Tasmania, Australia (Womersley, 1984; South & Skelton, 2003). As an invasive species in China, *C. valida* was first reported in sea cucumber aquaculture ponds in coastal provinces, including the Liaoning and Shandong Provinces (Chi et al., 2009; Deng et al., 2012). *C. valida*, a filamentous green tide forming algae, is visible throughout the year and dominates aquaculture ponds (Chi et al., 2009). *C. valida* grows more luxuriantly from April to September of every year, and sometimes the macroalgal mat exceeds 0.5 m in thickness. *C. valida* outbreaks present a significant problem in aquaculture ponds, especially when loose algal mats accumulate and decompose (Deng et al., 2012). A large number of *C. valida* floating on the surface of the water blocks the sunlight and suppresses the growth of benthic diatoms, which are the diet for sea cucumbers (Deng et al., 2012).

The growth and photosynthetic activity of algae are closely tied to the temperature, pH, salinity, and other environmental factors (Yun, Sangheon & Ikkyo, 2010; Fu, 2014). Although the growth of *Chaetomorpha sp*. is affected by the temperature and salinity of the culture environment, the algae can grow rapidly in stagnant water, despite large fluctuations in salt water and temperature (Tsutsui et al., 2015; Gao, Endo & Agatsuma, 2018a, 2018b). *Scrippsiella trochoidea* and *Alexandrium tamarense* are very sensitive to pH, with a suitable pH range of 7.0–9.0, and an optimal pH of 7.5–8.0 (Deng et al., 2004). Chen et al. (2015) found that the photosynthetic activity of *Ceratophyllum demersum* was higher between pH 7 and 9. With increasing salinity, temperature, and light intensity, the photosynthesis of *Chaetoceros aeruginosa* first increased and then decreased. When the salinity was more than 40 ‰, the photosynthesis of the *Chaetoceros aeruginosa* was inhibited (Qi et al., 2008). Thus, different environmental factors may promote the growth and photosynthesis of algae, resulting in its overgrowth and potential outbreak. However, very little research has been done on the *Chaetomorpha* (De Paula Silva et al., 2008; Xu & Lin, 2008; Deng et al., 2012).

The aim of this study was to clarify the ecological conditions for optimal growth of *C. valida* and evaluate its tolerance to pH and salinity in sea cucumber aquaculture ponds near the coast in northern China. The ultimate goal of the study was to provide an explanation of why *C. valida* outbreaks occur. To achieve this goal, we conducted both a field survey and laboratory studies to understand the effects of salinity and pH on *C. valida* growth.

## Materials and Methods

### Field surveys

Field surveys were carried out in the aquaculture ponds of sea cucumbers (36°46′N, 121°9′E). The aquaculture pond area was 80 acres. The biomass of sea cucumber was 300–400 kg/acre. Water was exchanged twice a month over a 3 to 4 day period, exchanging approximately 1/3 per day. Three ponds were selected randomly, and samples were collected at six randomly selected fixed sites in each pond. Each sampling site was separated into two m^2^ quadrats (1 × 1m). The samples were collected on the 15th of each month. Salinity and pH of the seawater in the ponds were recorded from January to December in 2016. Salinity was measured with a salinity meter (MASTER-S10α; Shanghai Renhe Scientific Instrument Co., Ltd., Shanghai, China), and pH was measured with a pH meter (PHS-3C; Shanghai INESA Instruments Co., Ltd., Shanghai, China).

*Chaetomorpha valida* samples were harvested from the sampling spot, other algae were removed, sediment was cleaned from the algae surface with clean seawater, and the sample was placed on a bamboo pole and air-dried for 10 min. The wet weight of the algae was checked every month. After recording the wet weight of *C. valida*, the algae were returned to the original cages to monitor the annual growth of algae. The last wet weight was measured on the shore and the *C. valida* was not returned to the ponds.

### Laboratory experiments

*Chaetomorpha valida* were collected from a sea cucumber aquaculture pond in October 2016 and transported in seawater to the laboratory. Healthy thalli, which were full of plump cells, were selected and cleaned with sterilized seawater to remove epiphytes and their metabolites. Then healthy thalli were cut into 2.5 cm fragments for the experiment, using a scale.

For the salinity experiments, seawater was filtered and sterilized. The salinity of seawater was diluted with deionized water to 22‰. Using sodium chloride, the salinity of seawater was adjusted to 22‰, 24‰, 28‰, 30‰, 32‰, 34‰, 36‰, and 40‰. A total of 30 fragments were placed in a 90 mm petri dish, and 15 mL of seawater was added to each dish. The petri dish was covered during the experiment. Samples were analyzed in triplicate for each salinity group. Samples were incubated for 18 days at 25 °C, with a light intensity of 108 mol photon·m^−2^·s^−1^, and a photoperiod of 12 L:12 D. Alga length was measured with a scale every 2 days. After 30 min of dark-adaptation, the *F*_v_/*F*_m_ (quantum yield of photosynthesis) of algae was measured with an AquaPen-P AP-P 100 (Photon Systems Instruments, Ltd., Brno, Czech Republic) at 4 and 18 days. Seawater with different salinity was changed every 2 days.

For the pH experiments, the pH of seawater was adjusted to 6.5, 7.0, 7.5, 8.0, and 8.5 with sodium hydroxide and hydrochloric acid. The seawater salinity was 32‰. Culture conditions and treatments were the same as described previously. Samples were analyzed in triplicate for each pH group. Algae length was measured with a scale every 2 days during the 32 days of the experiment. The *F*_v_/*F*_m_ of algae was measured with an AquaPen-P AP-P 100 (Photon Systems Instruments, Ltd., Brno, Czech Republic) every 2 days. Seawater with different pH was changed every 2 days.

### Data analysis

Data were processed using Origin 8.5. Values are reported as mean ± standard deviation. Regression analysis was used to analyze the relationship between various factors. Statistical analyses were one-way ANOVA with Dunnett’s posttest, and *p* < 0.05 was considered significant. These analyses were conducted using SPSS 13.0.

## Results

### Field surveys

**Figure 1:**
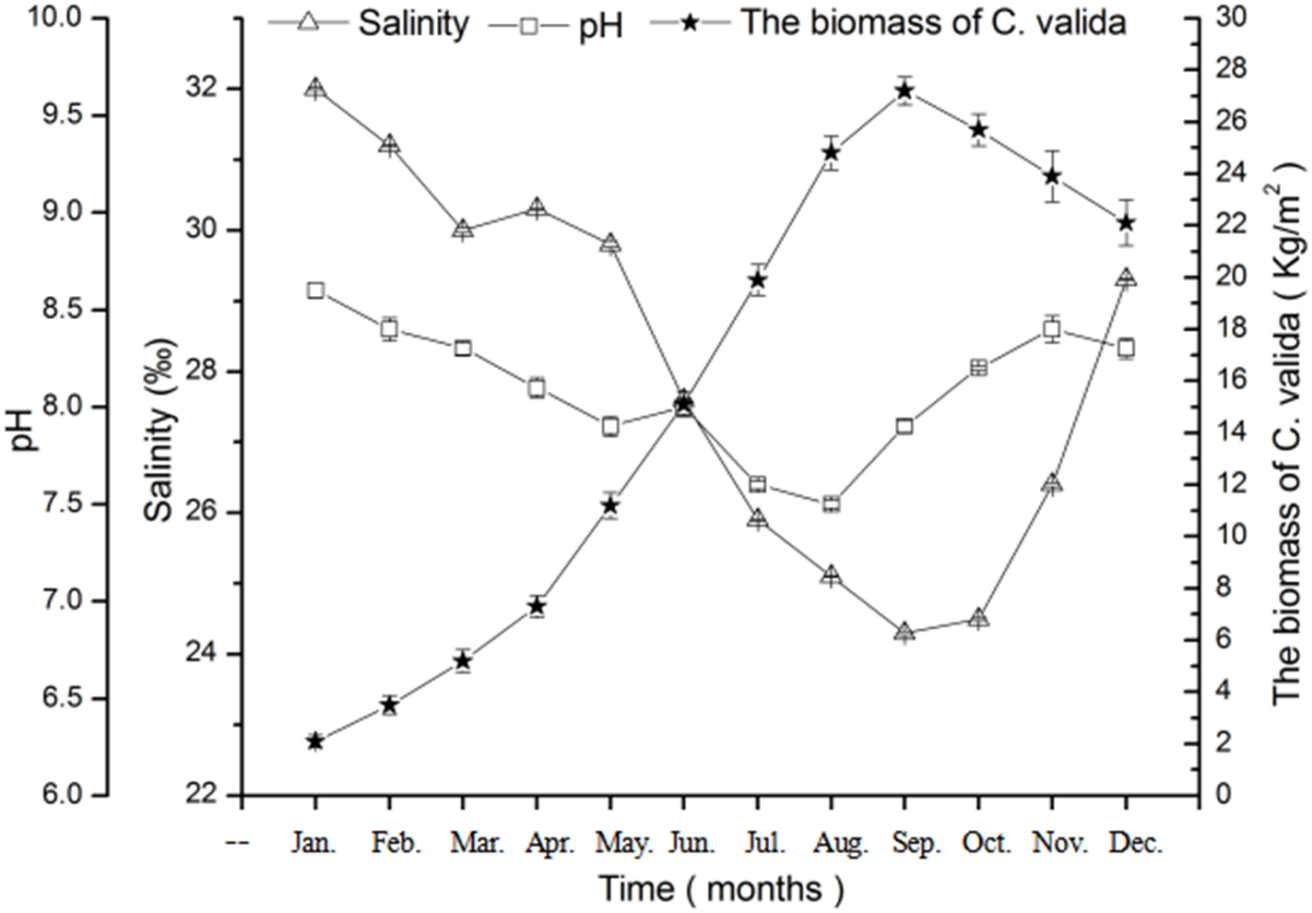
Changes in salinity, pH, and biomass of *C. valida* in ponds of sea cucumber over time. Values are expressed as mean ± standard deviation (*n* = 18).

The salinity changed significantly over time (*p* = 1 × 10^−6^, d*f* = 11, *F* = 74,642.628) (Fig. 1). Salinity varied from 24.3 ± 0.01‰ to 32.0 ± 0.02‰. As shown in Fig. 1, the salinity of seawater decreased from June to September (summer and early autumn) then increased from October to December (autumn and early winter), and salinity was lowest at the end of summer.

The results of the field survey show that pH changed significantly over time in the sea cucumber ponds (*p* = 1 × 10^−5^, d*f* = 11, *F* = 451.595) (Fig. 1). The pH tended to decrease first and then increase. The pH varied from 7.5 ± 0.02 to 8.6 ± 0.04.

The biomass of *C. valida* changed significantly over time (*p* = 1 × 10^−6^, d*f* = 11, *F* = 2,448.658) (Fig. 1). The biomass of *C. valida* increased first and then decreased later in the year. From January to May, the alga biomass was first low and then gradually increased. The biomass of *C. valida* peaked in September (27.2 ± 0.541 Kg/m^2^) and decreased gradually.

The results of the field investigation showed that the biomass of *C. valida* was inversely proportional to the salinity and pH of seawater in the ponds (Fig. 1). In the early stages, salinity and pH were high, at which time the biomass of *C. valida* was lower. Over time, salinity and pH decreased, while *C. valida* biomass increased gradually. Salinity and pH reached their lowest values in August and September, whereas the biomass of *C. valida* peaked in September. After peaking, the *C. valida* biomass gradually decreased while seawater salinity and pH increased rapidly.

### Laboratory experiments

**Figure 2:**
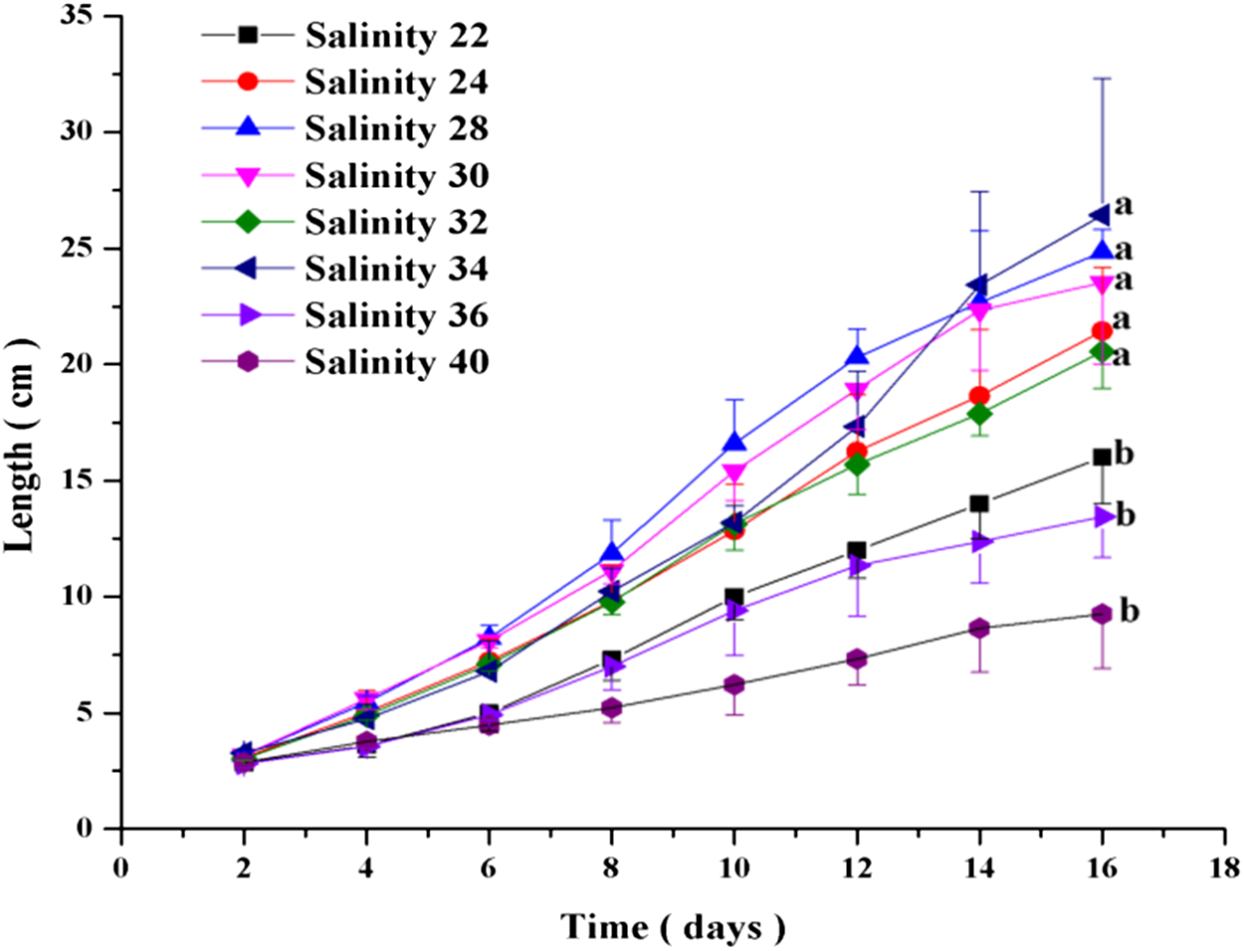
Effects of salinity on the growth of *C. valida.* Values are expressed as mean ± standard deviation; *n* = 3 replicates. The different letters on the right side of the curve indicate significant differences (one-way ANOVA with Dunnett’s posttest α = 0.05): a<b.

Salinity had significant effects on the growth of *C. valida* (*p* = 1 × 10^−4^, d*f* = 7, *F* = 12.205) (Fig. 2) on day 16. The growth of *C. valida* increased significantly when grown in salinity of 24–34‰ compared to the 22‰ salinity. However, no significant differences in *C. valida* growth were detected at 36‰ and 40‰ salinity compared to 22‰ salinity. At the same salinity, the length of *C. valida* increased over time. At the same culture time, the length of *C. valida* increased with increasing salinity, but then decreased at higher salinity. When the salinity was 40‰, the growth of *C. valida* was relatively low, and the length was only 9.26 ± 2.36 cm after 16 days in culture. In contrast, *C. valida* grew faster when the salinity was between 24‰ and 34‰. When the salinity was 34‰, the length of *C. valida* was 26.44 ± 5.89 cm after 16 days in culture, which was 10.6 times the initial value (day 2).

**Figure 3:**
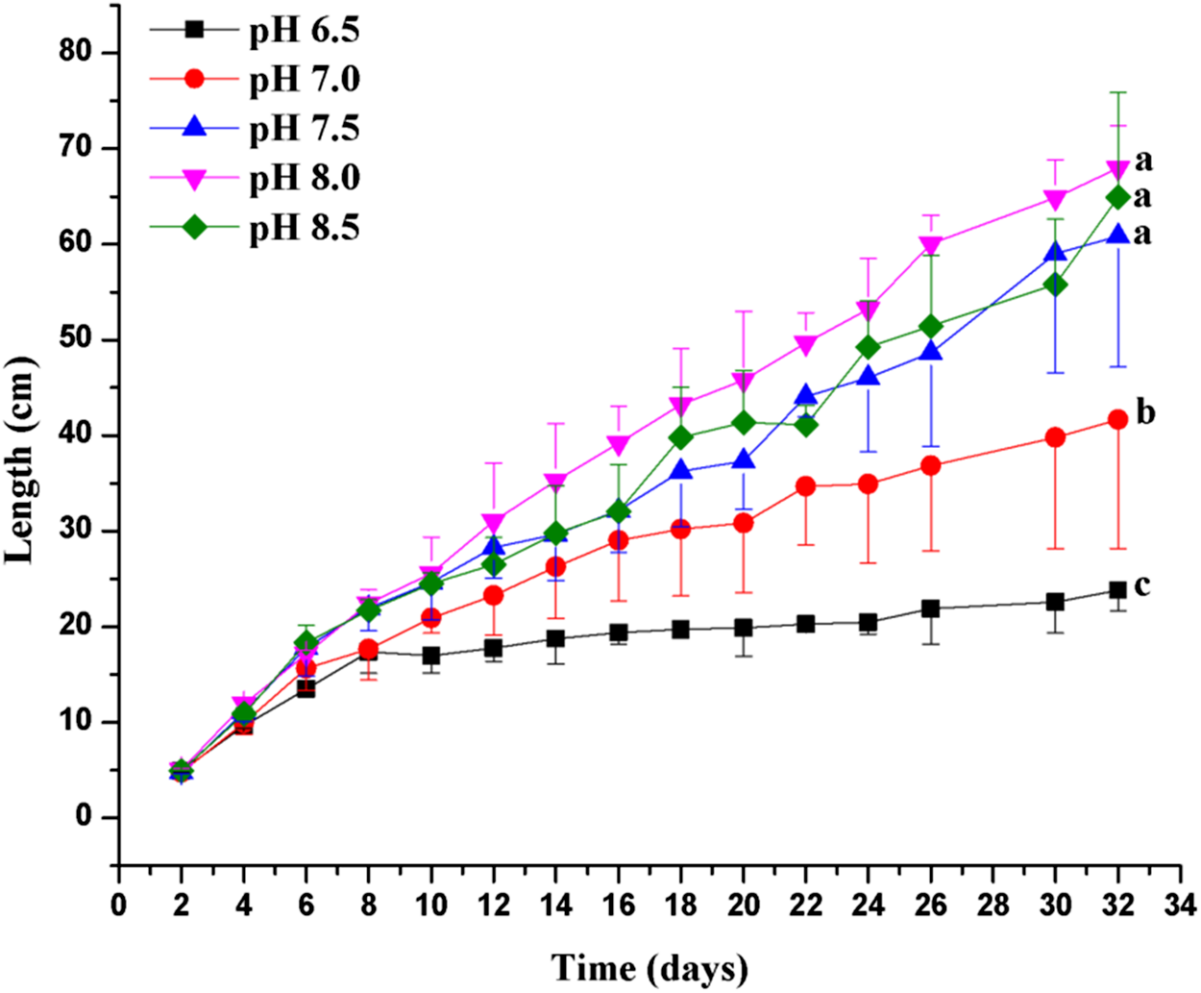
Effects of pH on the growth of *C. valida*. Values are expressed as mean ± standard deviation; *n* = 3 replicates. The different letters on the right side of the curve indicate significant differences (one-way ANOVA with Dunnett’s posttest α = 0.05): a<b<c.

The pH affected the grown of *C. valida* significantly (*p* = 0.001, d*f* = 4, *F* = 10.238) (Fig. 3). At pH 7.5–8.5, the growth of *C. valida* increased significantly compared to pH 6.5–7.0 (Fig. 3). At the same pH, the length of *C. valida* increased over time. At the same culture time, the length of *C. valida* increased with increasing pH and peaked at pH 8.0. At 32 days of culture, *C. valida* grew to 67.96 ± 4.45 cm at pH 8.0, which was 27.18 times the initial value. However, *C. valida* samples only grew to 23.83 ± 2.16 cm at a pH of 6.5.

**Figure 4:**
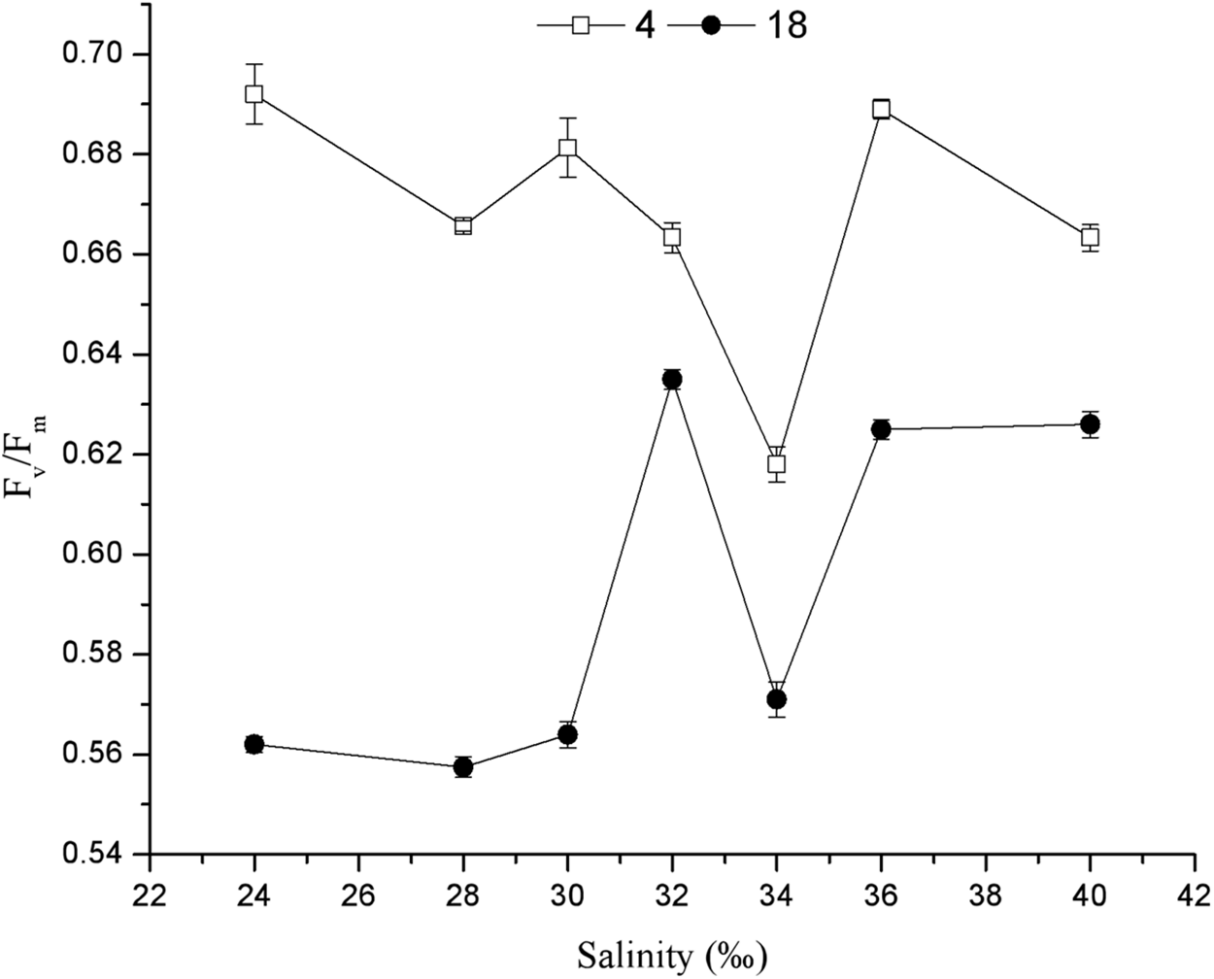
Effects of salinity on the *F*_v_/*F*_m_ of *C. valida* after 4 and 18 days of culture. Values are expressed as mean ± standard deviation; *n* = 3 replicates.

Salinity significantly affected the *F*_v_/*F*_m_ of *C. valida* (*p* = 1 × 10^−5^, d*f* = 6, *F* = 530.941) (Fig. 4). At 18 days in culture, the *F*_v_/*F*_m_ of *C. valida* increased with increasing salinity. When the salinity was 32‰, *F*_v_/*F*_m_ was the highest (0.635 ± 0.002). Under the same salinity conditions, *F*_v_/*F*_m_ of *C. valida* decreased significantly with time (4 days vs. 18 days). At 4 days in culture, *F*_v_/*F*_m_ was the lowest (0.620 ± 0.004) when the salinity was 34‰.

**Figure 5:**
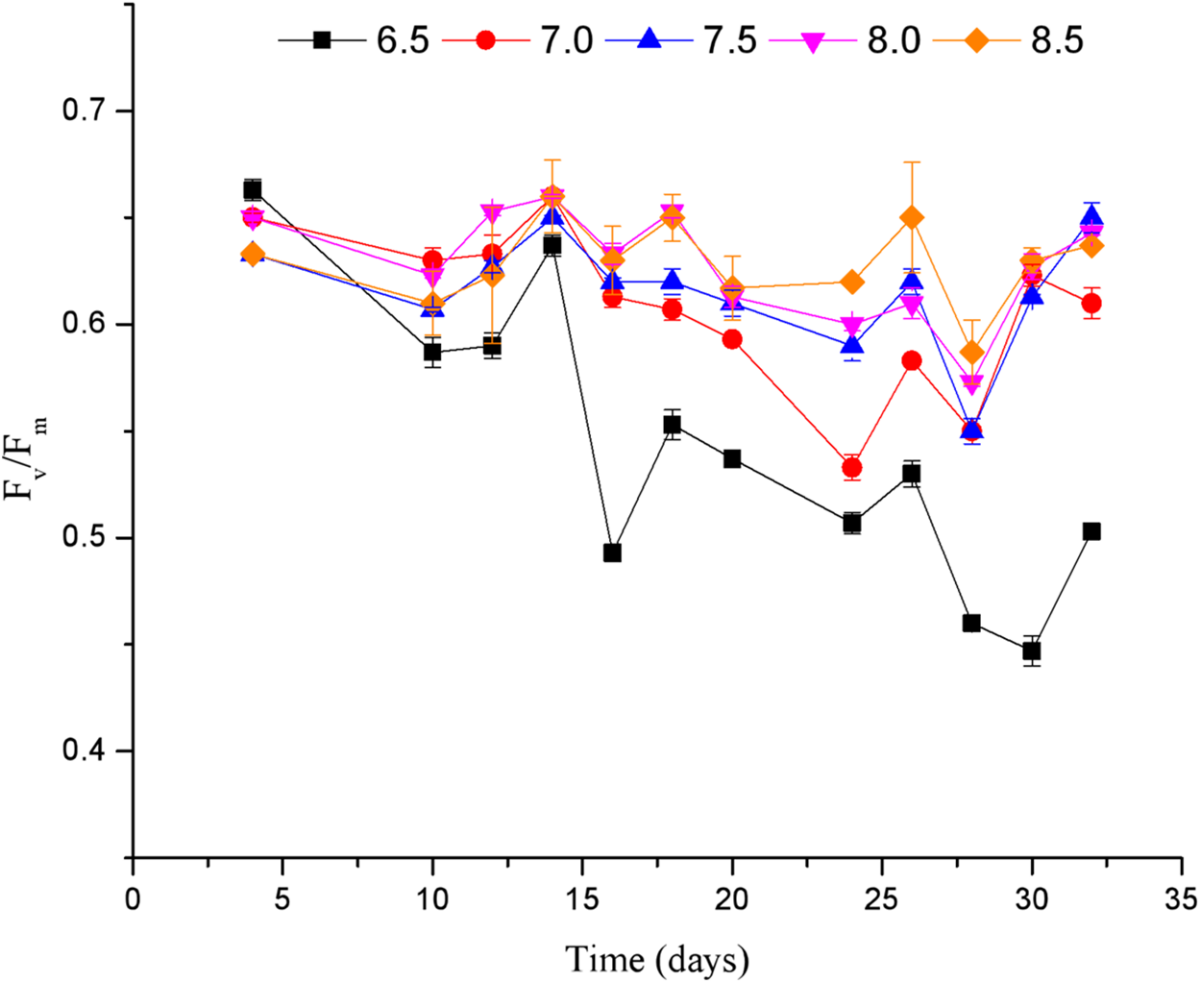
Effects of pH on the *F*_v_/*F*_m_ of *C. valida*. Values are expressed as mean ± standard deviation; *n* = 3 replicates.

The pH had significant effects on the *F*_v_/*F*_m_ of *C. valida* (*p* = 1 × 10^−5^, d*f* = 4, *F* = 520.696) (Fig. 5). At the same pH, *F*_v_/*F*_m_ of *C. valida* was unstable over time. When the pH was 6.5, *F*_v_/*F*_m_ was lower compared to other pH conditions (0.50 ± 0.006 vs. 0.63 ± 0.001, pH 6.5 vs. pH 8 on day 32). *F_v_/F_m_* ranged from 0.55 ± 0.006 to 0.65 ± 0.007 at pH 7.5.

## Discussion

### Field survey

In the field survey, we found that salinity, pH, and biomass of *C. valida* in the ponds fluctuated. In summer and autumn, the salinity and pH of the seawater decreased, while the biomass of the *C. valida* increased rapidly. We speculate that the root cause for the changes in salinity and pH were more rainfall in summer and autumn. In sea cucumber ponds, sediments, seawater, and organisms constitute a complete ecological system, and this system has a self-regulating effect; the environment affects algae growth and the algae growth affects the environment (Tang et al., 2013). During summer and autumn, the biomass of *C. valida* was highest, and *C. valida* even bloomed, which may have been due to changes in the salinity and pH of seawater.

The results of the field investigation showed that the biomass of *C. valida* was inversely proportional to the salinity and pH of seawater in the ponds. In the early stages, salinity and pH were higher, at which time the biomass of *C. valida* was low. As time progressed, salinity and pH decreased, while the biomass of *C. valida* increased rapidly. Salinity and pH reached their lowest values in September and August, respectively, whereas the biomass of *C. valida* peaked in September. In subsequent months, a rapid increase in seawater salinity and pH was accompanied by decreases in *C. valida* biomass. These observations show that the algae grew even before the ecological environment was most suitable for their rapid growth, and the previous environmental conditions may have provided the impetus for their rapid growth. In the ecosystem of sea cucumber aquaculture ponds, the high salinity and the high pH during the period of low biomass of *C. valida* may have laid the foundation for rapid growth in the next few months. In addition to the changes in pH and salinity, the increase in temperature during the summer months may have further accelerated the growth of *C. valida.* Many factors interact in the field environment, and it is difficult to isolate the effects of pH and salinity in the field, so laboratory experiments were conducted to verify the conclusions from the field experiments.

### Static simulation in the laboratory

Salinity and pH are environmental factors that affect the growth of *C. valida*. Seawater salinity varies depending on the location of the sea, which affects the climate (Lin et al., 2005). The main factors affecting salinity are precipitation and evaporation. Marine life living in estuaries is exposed to rapid changes in salinity and temperature, and these organisms generally have a wide range of salinity and temperature tolerance (Matsuyamaserisawa, Serisawa & Tanaka, 2004; Tsutsui et al., 2015). Tsutsui et al. (2015) found that *Chaetomorpha sp.* survived in salinities of 3.4–90.0‰. However, we found that when the salinity was 40%, the growth of *C. valida* was very low, and the length of the *C. valida* was relatively short. According to our data, the growth of *C. valida* was more rapid at salinities between 24‰ and 34‰ compared to other salinities. This result is in agreement with the field investigation, indicating that *C. valida* can grow rapidly in relatively high salinity, just like the salinity in sea cucumber ponds from January to May.

One of the most important physical and chemical parameters of aqueous solutions is pH, which is a measure of hydrogen ion concentration and is often referred to as the acidity and alkalinity of a solution. We showed that the growth of *C. valida* increased with increasing pH. According to our data, *C. valida* growth increased significantly at pH 7.5–8.5 compared to pH 6.5 and 7.0. This result indicates that *C. valida* can grow rapidly at the higher pH of the sea cucumber culture environment. However, algae have a strong buffering capacity for pH, possibly through carbon dioxide and nutrient metabolism. Thus, the proliferative activity of some algae species can change the pH of the water (Xu et al., 2009). The results of this experiment show that pH had an effect on the growth of *C. valida*, and *C. valida* blooms may change the salinity and pH of the seawater.

*F*_v_/*F*_m_ is the maximum photochemical efficiency of Photosynthetic system II (PSII) in the dark or the maximal quantum yield of PSII. *F*_v_/*F*_m_ reflects the intrinsic light intensity conversion efficiency (PSII), or the largest PSII light energy conversion efficiency (maximal PSII efficiency). Changes in *F*_v_/*F*_m_ during normal growth of algae are very small and are not affected by the species and growth conditions. However, environmental stress conditions result in decreased *F*_v_/*F*_m_. The results of this study demonstrate that the *F*_v_/*F*_m_ is maintained between 0.5 and 0.7 while *C. valida* is exposed to a wide range of salinity. When salinity was between 24% and 40%, *C. valida* maintained normal photosynthesis (*F*_v_/*F*_m_ between 0.56 ± 0.003 and 0.69 ± 0.002). In contrast, when the pH was 6.5, the *F*_v_/*F*_m_ value of *C. valida* eventually fell below 0.50, indicating that the photosynthesis of the *C. valida* was weak and not conducive to the growth of the algae. Under other pH conditions, the *F*_v_/*F*_m_ value of *C. valida* was maintained at about 0.6, indicating that the photosynthesis of *C. valida* was stronger and favorable for the growth of algae. Many reports show that salinity and pH affect the photosynthesis of algae (Borlongan et al., 2016). The results of this experiment show that *C. valida* is adaptable to the pH in the sea cucumber pond environment.

There are many factors affecting the growth of *Chaetomorpha* including temperature, light, nutrients, salinity, pH, and CO_2_ (Gao, Endo & Agatsuma, 2018a, 2018b). The reported factors affecting the growth of *C. valida* are temperature and light (Deng et al., 2012). In the course of our research, we found that in addition to temperature and light, salinity and pH had a significant effect on the growth of *C. valida.* Other factors will be studied in subsequent research, including nutrients and sediment in sea cucumber ponds.

## Conclusion

In a field survey, we demonstrate that the growth of *C. valida*, a species of filamentous green tide macroalgae, is closely related to the salinity and pH in the seawater from ponds in which sea cucumber are grown. The high salinity and the high pH during the period of low biomass of *C. valida* may lay the foundation for its rapid growth in the next few months. The results of laboratory experiments verified that salinity and pH were the two main factors that affected the growth of *C. valida*. At the same time, the results show that *C. valida* can bloom in sea cucumber ponds despite the relatively high salinity and high pH compared to coastal sea water.

## Supplemental Information

10.7717/peerj.6468/supp-1 Supplemental Information 1 Field survey raw data and data analysis.

10.7717/peerj.6468/supp-2 Supplemental Information 2 Lab experiment raw data and data analysis.

## Additional Information and Declarations

### Data Availability

The following information was supplied regarding data availability: The raw data are available in the Supplemental Files, results, and figures.
